# Prescribing Patterns of Oral Antibiotics and Isotretinoin for Acne in a Colorado Hospital System: Retrospective Cohort Study

**DOI:** 10.2196/42883

**Published:** 2023-08-21

**Authors:** Madeline J Adelman, Torunn E Sivesind, Isaac Weber, Grace Bosma, Camille Hochheimer, Chante Karimkhani, Lisa M Schilling, John S Barbieri, Robert P Dellavalle

**Affiliations:** 1 Department of Dermatology University of Colorado Anschutz Medical Campus Aurora, MI United States; 2 School of Medicine University of Missouri Columbia, MO United States; 3 Department of Biostatistics and Informatics University of Colorado School of Public Health Aurora, CO United States; 4 Flatirons Dermatology Broomfield, CO United States; 5 Department of Medicine and Division of General Internal Medicine University of Colorado Anschutz Medical Campus Aurora, CO United States; 6 Department of Dermatology Brigham and Women’s Hospital Boston, MA United States; 7 Department of Dermatology Rocky Mountain Regional Veterans Affairs Medical Center Aurora, CO United States; 8 Department of Epidemiology Colorado School of Public Health Aurora, CO United States

**Keywords:** acne, antibiotics, databases, guidelines, isotretinoin, prescribing, retinoids

## Abstract

**Background:**

Guidelines established by the American Academy of Dermatology recommend oral antibiotics as first-line therapy for mild, moderate, and severe acne. However, it is recommended to minimize the duration of oral antibiotic use, and there is increasing support for other systemic agents for acne.

**Objective:**

We sought to characterize the use of oral antibiotics and isotretinoin for the treatment of acne in the pediatric and young adult population aged 10 through 20 years and the adult population aged 21 to 45 years from 2011 to 2019.

**Methods:**

We conducted a retrospective, observational cohort study using electronic data from the enterprise data warehouse of the University of Colorado Anschutz Medical Campus and its affiliates, with data in the format of the Observational Health Data Sciences and Informatics (OHDSI) Observational Medical Outcomes Partnership (OMOP) common data model. Categorical values (sex, race, and ethnicity) were compared using chi-square tests, and continuous variables (age) were compared using 2-tailed *t* tests.

**Results:**

Our cohort of 15,704 patients was composed of mostly White (12,776/15,704, 81.4%), non-Hispanic or Latino (13,307/15,704, 84.7%), and female (11,093/15,704, 70.6%) patients. Among the 4605 male patients in the eligible cohort, 1810 (39%) received an oral antibiotic treatment, in comparison to 3109 (28%) of the 11,093 eligible women (*P*<.001). Among the 4605 men who were eligible for treatment with isotretinoin in this population, 988 (21.5%) received a course of isotretinoin, compared to only 10.4% (1159/11,093) eligible women (*P*<.001). Male patients were 1.67 times more likely to have received an antibiotic prescription (odds ratio [OR] 1.67, 95% CI 1.55-1.79) and over twice as likely to have received an isotretinoin prescription (OR 2.34, 95% CI 2.13-2.57) than female patients.

**Conclusions:**

Minocycline was the most frequently prescribed antibiotic for the treatment of acne in this study cohort. From 2015 to 2019, there was no significant change in the number of antibiotic prescriptions over time. Men were significantly more likely to receive both oral antibiotics and isotretinoin than female patients. Multiple factors could be contributing to this discrepancy, including the burden of iPLEDGE, additional systemic treatment options for female patients, and the difference in acne severity across sexes. We could not determine the difference in severity of acne between male and female patients in our cohort, and further research is needed to ascertain the variation across sexes.

## Introduction

Acne is a common and debilitating medical condition, particularly among adolescents and young adults [1-3]. Guidelines established by the American Academy of Dermatology (AAD) recommend systemic antibiotics as first-line treatment for mild, moderate, and severe inflammatory acne, in combination with topical retinoids and benzoyl peroxide [1]. Monotherapy with systemic antibiotics is not recommended, and it is suggested that patients be re-evaluated every 3 to 4 months to limit antibiotic use to the shortest duration possible to avoid bacterial resistance [1]. In addition to promoting resistance, long-term oral antibiotic use has been associated with a number of adverse events, including microbiome disruption and pharyngitis, as well as possible associations with inflammatory bowel disease (IBD) and obesity [4,5]. Several studies have analyzed the prescription patterns of acne treatments [4-9]. This study investigated oral antibiotic and isotretinoin use for acne in a Colorado hospital system between 2011 and 2019.

## Methods

### Data Source

We performed a retrospective, observational cohort study of patients treated for acne using electronic data from Health Data Compass, the enterprise data warehouse of the University of Colorado Anschutz Medical Campus and its affiliates, and from Children’s Hospital Colorado [10]. Data were obtained in the format of the Observational Medical Outcomes Partnership (OMOP) common data model from the Observational Health Data Sciences and Informatics (OHDSI) network. OHDSI is an interdisciplinary collaborative composed of an international network of researchers and health databases. The OMOP common data model was chosen for this study to facilitate future collaboration and expansion of this study to other sites within the OHDSI network and because of its methods library, which facilitates both large-scale implementation of observational study designs and large-scale data analytics. The cohort and outcome phenotype code and analytic code are available on GitHub [11].

### Study Design and Study Population

Outcomes of interest were the type of oral antibiotic therapy prescribed for patients with acne diagnoses. All study subjects were aged between 10 and 45 years as of January 1, 2015, with a diagnosis of acne defined by at least 2 diagnoses of acne, as represented by select OHDSI concept IDs (Multimedia Appendix 1). While a variety of acne diagnoses fell into our inclusion criteria phenotype, only the following appeared in our population: acne, acne conglobata, acne varioliformis, excoriated acne, and tropical acne. Antibiotic start date was on or after January 1, 2015, occurring on or after any diagnosis of acne, with at least one year of follow-up from the first oral antibiotic prescription index date. Prescriptions for oral liquid or suspension forms were excluded. Similar data were collected regarding prescriptions for isotretinoin.

In an attempt to exclude antibiotics prescribed for reasons other than acne, the minimum antibiotic dose required was a prescription quantity of 28 or more, unless determined by the physician authors (MJA, TES) consensus to represent an equivalent quantity (eg, prescriptions for packs containing multiple doses).

### Statistical Analysis

We assessed the prevalence of antibiotics and isotretinoin prescriptions for the treatment of acne according to age and sex. Categorical values (sex, race, and ethnicity) were compared using chi-square tests, and continuous variables (age) were compared using 2-tailed *t* tests. All tests were evaluated at the *P*=.05 significance level. R (version 3.6.0; R Core Team) was used for all summaries and figures. We assessed whether the proportion of patients who received medication was different based on sex using an unadjusted logistic regression model with the binary outcome of receiving or not receiving medication.

### Ethical Considerations

This study is exempt from institutional review board assessment under category four of the University of Colorado Exempt Research Guidelines [12].

## Results

Among a total of 15,704 patients who were identified as meeting the inclusion and exclusion criteria for our outcome of interest, a total of 4920 patients received antibiotic prescriptions (Table 1).

Our cohort had a mean age of 22.3 (SD 8.6) years, and was composed of mostly (12,776/15,704, 81.4%), non-Hispanic or Latino (13,307/15,704, 84.7%), and female (11,093/15,704, 70.6%) patients (Table 1). The most common diagnosis was acne: International Statistical Classification of Diseases and Related Health Problems (ICD) code 1569798 (15,668/15,704 99.8%).

There were statistically significant differences in age, sex, and race when comparing those who did and did not receive an antibiotic prescription; however, the magnitude of the differences in age and race was very small and not clinically meaningful (Table 1). Of the patients who received antibiotics, 63.2% (3109/4920) were female. However, men were more likely than women to receive a prescription for antibiotics (1810/4605, 39% vs 3109/11,093, 28%; *P*<.001).

Findings were similar when comparing those who did and did not receive an isotretinoin prescription. Differences in age, sex, and race were all statistically significant, but only sex was associated with a clinically meaningful difference between patients with and without isotretinoin treatment (Table 2). Men were more likely than women to receive a prescription for isotretinoin (988/4605, 21.5% vs 1159/11,093, 10.4%; *P*<.001).

Using an unadjusted logistic regression model with the binary outcome of receiving medication or not, we were able to assess the odds of receiving antibiotics or isotretinoin based on sex (Table 3). Our results suggest that male patients were 1.67 times more likely to have received an antibiotic prescription than female patients (odds ratio [OR] 1.67, 95% CI 1.55-1.79) and over twice as likely (OR 2.34, 95% CI 2.13-2.57) to have received an isotretinoin prescription than female patients.

**Figure 1:**
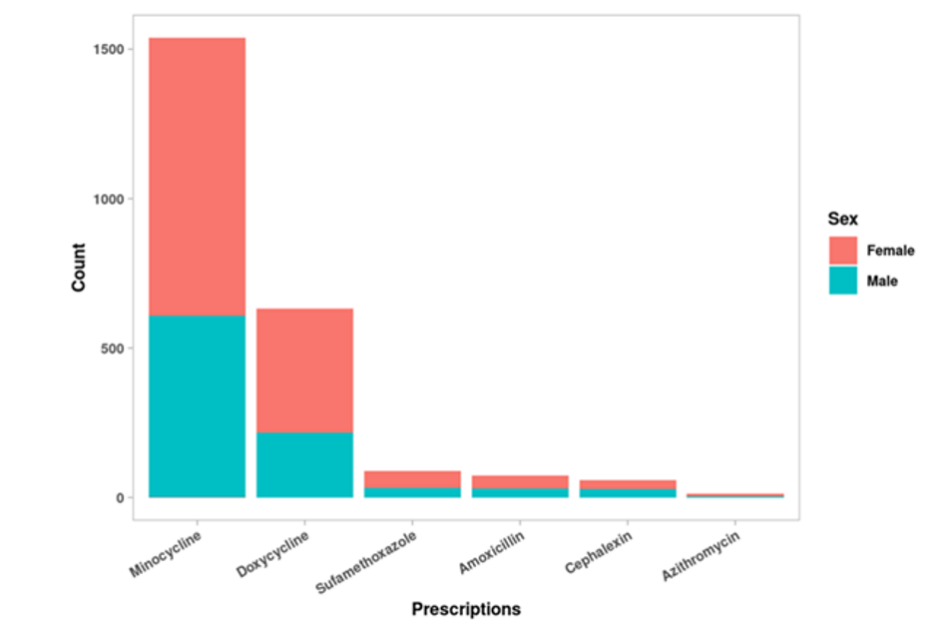
Frequency of prescription for each antibiotic.

**Figure 2:**
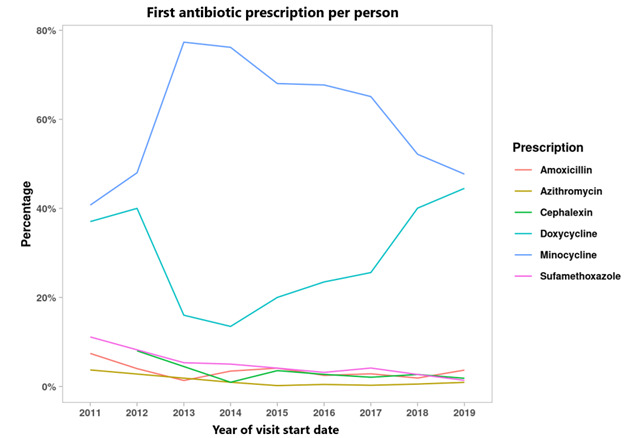
First antibiotic prescription over time by proportion.

The most prescribed antibiotic was minocycline, followed by doxycycline (Figure 1). Minocycline was also the most prevalent initial antibiotic prescribed for every year captured in this analysis, followed by doxycycline (Figure 2). Figure 2 also illustrates a slow rise in doxycycline prescription prevalence starting in 2013 and a slow decline in minocycline prescriptions starting in 2013. Some antibiotic prescriptions may have been missed, particularly in the years 2011-2014, where the total count of antibiotics for these data points was much lower than in the period 2015-2019 (Table 4). However, when looking at the total amount of antibiotic prescriptions over time from 2015-2019, there is no significant observable change in the use of oral antibiotics over this time period (Table 4).

**Table 1:** Cohort demographics.

User prevalence by antibiotics			No antibiotic prescribed (n=10,784)		Antibiotic prescribed (n=4920)		All participants (N=15,704)
Age (years), mean (SD)			22.9 (8.5)		21.1 (8.5)		22.3 (8.6)
**Sex, n (%)**							
	Female	7984 (74)		3109 (63.2)		11,093 (70.6)	
	Male	2795 (25.9)		1810 (36.8)		4605 (29.3)	
	Missing	5 (0)		1 (0)		6 (0)	
**Race, n (%)**							
	American Indian or Alaska Native	39 (0.4)		15 (0.3)		54 (0.3)	
	Asian	305 (2.8)		108 (2.2)		413 (2.6)	
	Black or African American	291 (2.7)		93 (1.9)		384 (2.4)	
	Native Hawaiian or Other Pacific Islander	17 (0.2)		8 (0.2)		25 (0.2)	
	White	8708 (80.7)		4068 (82.7)		12,776 (81.4)	
	Missing	1424 (13.2)		628 (12.8)		2052 (13.1)	
**Ethnicity, n (%)**							
	Hispanic or Latino	920 (8.5)		417 (8.5)		1337 (8.5)	
	Non-Hispanic or Latino	9195 (85.3)		4112 (83.6)		13,307 (84.7)	
	Missing	669 (6.2)		391 (7.9)		1060 (6.7)	
**Diagnosis, n (%)**							
	Acne	10,761 (99.8)		4907 (99.7)		15,668 (99.8)	
	Acne conglobate	6 (0.1)		2 (0)		8 (0.1)	
	Acne varioliformis	5 (0)		6 (0.1)		11 (0.1)	
	Excoriated acne	9 (0.1)		4 (0.1)		13 (0.1)	
	Tropical acne	3 (0)		1 (0)		4 (0)	

**Table 2:** User prevalence among isotretinoin recipients.

User prevalence by isotretinoin		None (n=13,555)	Received medication (n=2148)
Age (years), mean (SD)		22.8 (8.7)	19.6 (6.6)
**Sex, n (%)**			
	Female	9934 (73.3)	1159 (54)
	Male	3617 (26.7)	988 (46)
	Missing	5 (0)	1 (0)
**Race, n (%)**			
	American Indian or Alaska Native	51 (0)	3 (0.1)
	Asian	373 (2.8)	40 (1.9)
	Black or African American	349 (2.6)	35 (1.6)
	Native Hawaiian or Other Pacific Islander	23 (0.2)	2 (0.1)
	White	11,010 (81.2)	1766 (82.2)
	Missing	1750 (12.9)	302 (14.1)
**Ethnicity, n (%)**			
	Hispanic or Latino	1182 (8.7)	155 (7.2)
	Non-Hispanic or Latino	11,521 (85)	1786 (83.1)
	Missing	853 (6.3)	207 (9.6)
**Diagnosis, n (%)**			
	Acne	13,524 (99.8)	2144 (99.8)
	Acne conglobata	5 (0)	3 (0.1)
	Acne varioliformis	10 (0.1)	1 (0)
	Excoriated acne	13 (0.1)	0 (0)
	Tropical acne	4 (0)	0 (0)

**Table 3:** Odds ratio of receiving medication by sex.

Outcome	Odds ratio^a^ (CI)	*P* value
Antibiotics	1.7 (1.5-1.8)	<.001
Isotretinoin	2.3 (2.1-2.6)	<.001

^a^Reference level is female.

**Table 4:** Antibiotic prescriptions over time. Some antibiotic prescriptions may have been missed, particularly in the years 2011-2014.

Year	Prescriptions, n
2011	71
2012	136
2013	242
2014	735
2015	1763
2016	1867
2017	1781
2018	1808
2019	1509

## Discussion

### Overview

We characterized oral antibiotic use for acne treatment in a Colorado hospital system. Among 15,704 patients, we found that male patients were more likely than female patients to receive both antibiotic and isotretinoin prescriptions for acne. Among the eligible recipients of prescriptions for acne, there were 11,093 eligible female patients (70.6%) and 4605 eligible male patients (29.3%). However, among recipients of antibiotics, only 28% (3109/11,093) of female patients received prescriptions, compared to 39% (1810/4605) of male patients. Similarly, among recipients of isotretinoin prescriptions, only 10.4% (1159/11,093) of eligible female patients received prescriptions, compared to 21.5% (988/4605) of male patients. Male patients were 1.67 times as likely to receive antibiotics for acne and over twice as likely to receive isotretinoin for acne compared to female patients.

Previous research on the health burden of acne has revealed that women with acne demonstrate greater self-consciousness of their appearances and are more likely to seek care due to an increased subjective rating of severity [13]. Thus, female patients may seek care for less severe forms of acne compared to male patients, leading to a lower likelihood of being prescribed systemic medications for acne. Another potential explanation for this discrepancy could be that female patients have access to other systemic acne treatment options that male patients do not, namely, oral contraceptive pills (OCPs) and spironolactone [1,14-16]. This study did not include an analysis of antiandrogen medication.

Other studies suggest acne severity could be significantly higher in male patients, thus necessitating the need for systemic treatment [17]. However, further research is needed regarding the severity of acne presentations in male versus female patients, as we did not have this data and cannot say for certain that the female patients in our cohort had a less severe presentation compared to male patients.

The discrepancy in isotretinoin prescriptions may also reflect the increased burden of iPLEDGE, an FDA risk management program, on women capable of bearing children. In its initial introduction, iPLEDGE was associated with an initial 30% decrease in isotretinoin prescriptions for both men and women [18]. The system has requirements for patients, prescribers, pharmacies, and wholesalers. For women looking to start isotretinoin, iPLEDGE requires 2 negative pregnancy tests before starting therapy, monthly pregnancy tests during therapy, use of 2 forms of contraception during therapy, and use of 2 forms of contraception the month before and after therapy [19,20]. A 2013 pharmacy prescription claims–based study found that the overall number of isotretinoin prescriptions across all ages and sexes decreased after iPLEDGE implementation, with a greater decrease among women [19]. The extra requirements of iPLEDGE for women could be a dissuading factor, as they may be unable or unwilling to comply with the stringent contraception and testing requirements or may be considering pregnancy.

This study builds upon previous literature analyzing trends in antibiotic and isotretinoin prescriptions in dermatology (Table 5). In a large, retrospective study using OptumInsight data from 2004 to 2013, authors looked at prescribing patterns among dermatologists and nondermatologists for the treatment of acne [21]. They found a significant increase in spironolactone prescriptions for acne throughout that time period [21]. A separate retrospective OptumInsight study using data from 2007 to 2017 aimed to identify potential disparities in acne treatment and found that female patients were less likely than male patients to be prescribed both isotretinoin and oral antibiotics [6]. In addition, a retrospective population-based cohort study in British Columbia also found that individuals treated with isotretinoin were more likely to be male [5].

Minocycline was the most prescribed initial antibiotic and the most prevalent antibiotic in every year of data captured in this analysis (Figures 1 and 2). Beginning in 2013, doxycycline, the second most prevalent initial antibiotic, began a slow increase in prevalence, while minocycline prevalence began slowly declining. It is not completely clear why this trend occurred, as there is no overall clinical difference in efficacy between minocycline and doxycycline [22]. Side effects, such as an increased risk of lupus erythematous with minocycline, and patient and physician perceptions regarding the costs of the 2 drugs may have affected prescription patterns [22-25].

**Table 5:** Previous studies evaluating oral antibiotics and isotretinoin prescription patterns.

Studies	Total participants, N	Age (years)	Location	Period	Oral antibiotics OR^a,b^ (95% CI)	Oral antibiotics male to female ratio	Isotretinoin OR^a^ (), 95% CI	Isotretinoin male to female ratio	Duration of oral antibiotic therapy (days), mean (type of prescriber)
This study	15,700	22.3	Colorado, United States	2011-2019	1.67 (1.55-1.79)	1.39	2.34 (2.13-2.57)	2.14	—^c^
Barbieri et al [4]	79,600	12-22	United Kingdom	2003-2013	—	—	—	—	Nondermatologist: 175
Alhusayen et al [5]	1,500,000^d^	12-29	British Columbia, Canada	1997-2008	—	—	—	1.17	—
Barbieri et al [6]	30,000	15-35	United States	2007-2017	1.12 (1.06-1.18)	1.93	2.44 (2.10-2.95)	4.33	—
Barbieri et al [7]	572,600	15-27	United States	2004-2014	—	—	—	—	Dermatologist: 192; nondermatologist: 213
Straight et al [21]	16,500	32.6	United States	2008-2010	—	—	—	—	Dermatologist: 122; nondermatologist: 134

^a^Reference level is female.

^b^OR: odds ratio.

^c^Not available.

^d^Population based cohort (participants not confined to patients diagnosed with acne).

In contrast to this study, a cross-sectional analysis of antibiotic prescribing patterns among dermatologists from 2008 to 2016 found a decrease in antibiotic prescriptions for acne during that time [8]. We found no significant change in antibiotic prescription frequency in our cohort.

### Limitations

We used ICD codes, established by the Global Burden of Disease Study and validated by an international panel of skin disease experts, to determine the appropriate OHDSI acne concept IDs [26]. Some diagnoses may have been missed or inappropriately classified and were therefore not included in this study.

Similarly, some antibiotic prescriptions may have been missed, particularly in the years 2011-2014, where the total count of antibiotics for these data points was much lower than in the period 2015-2019. We used antibiotics cited in previous acne literature, and antibiotics not commonly prescribed for the treatment of acne or that are available over the counter were not captured [4,21]. Patients prescribed antibiotics for acne without an acne diagnosis would also have been missed, as would patients who were given a prescription for acne for a shorter time period than 28 days. We could also have potentially included patients who were on long-term antibiotics or isotretinoin for a reason other than acne, despite having 2 diagnoses of acne. Other data limitations include missing information on insurance, severity of disease, treatment duration, and prescription refills.

### Conclusions

In our sample, male patients were 1.67 times more likely to have received an antibiotic prescription and over twice as likely to have received an isotretinoin prescription than were female patients. These findings could be attributed to the fact that female patients may seek treatment for less severe acne, resulting in fewer systemic therapy prescriptions. Further research is needed regarding the severity of acne presentations in male versus female patients, as we did not have this data and cannot say for certain that the female patients in our cohort had a less severe presentation compared to male patients. Side effect profiles and additional systemic treatment options for male patients (OCPs and spironolactone) may also play a role in this discrepancy between sexes.

## Appendix

Multimedia Appendix 1 Acne diagnosis concept set.

## Abbreviations

AAD: American Academy of Dermatology
OHDSI: Observational Health Data Sciences and Informatics
OMOP: Observational Medical Outcomes Partnership
IBD: inflammatory bowel disease
ICD: International Statistical Classification of Diseases and Related Health Problems
OCP: oral contraceptive pill
