# Supplementary material for: Prescribing Patterns of Oral Antibiotics and Isotretinoin for Acne in a Colorado Hospital System: Retrospective Cohort Study
Source: JMIR Dermatol. 2023 Aug 21;6:e42883. doi: 10.2196/42883 (PMC10477922; doi:10.2196/42883)
Supplement: Multimedia Appendix 1 [file derma_v6i1e42883_app1.docx]

**Appendix**:

Acne diagnosis concept set.

| **Concept ID** | **Concept Code** | **Concept Name** | **Class** | **Domain** | **Vocabulary** |
| --- | --- | --- | --- | --- | --- |
| 44836157 | 706.1 | Other acne | 4-dig billing code | Non-Standard | Condition |
| 44824484 | 706 | Acne varioliformis | 4-dig billing code | Non-Standard | Condition |
| 35208638 | L73.0 | Acne keloid | 4-char billing code | Non-Standard | Condition |
| 35208628 | L70.9 | Acne, unspecified | 4-char billing code | Non-Standard | Condition |
| 35208627 | L70.8 | Other acne | 4-char billing code | Non-Standard | Condition |
| 35208626 | L70.5 | Acne excoriee | 4-char billing code | Non-Standard | Condition |
| 35208625 | L70.4 | Infantile acne | 4-char billing code | Non-Standard | Condition |
| 35208624 | L70.3 | Acne tropica | 4-char billing code | Non-Standard | Condition |
| 35208623 | L70.2 | Acne varioliformis | 4-char billing code | Non-Standard | Condition |
| 35208622 | L70.1 | Acne conglobata | 4-char billing code | Non-Standard | Condition |
| 35208621 | L70.0 | Acne vulgaris | 4-char billing code | Non-Standard | Condition |
| 1569798 | L70 | Acne | 3-char nonbill code | Non-Standard | Condition |
